# Experience Modulates Vicarious Freezing in Rats: A Model for Empathy

**DOI:** 10.1371/journal.pone.0021855

**Published:** 2011-07-13

**Authors:** Piray Atsak, Marie Orre, Petra Bakker, Leonardo Cerliani, Benno Roozendaal, Valeria Gazzola, Marta Moita, Christian Keysers

**Affiliations:** 1 Department of Neuroscience, University Medical Center Groningen, Groningen, The Netherlands; 2 Netherlands Institute for Neuroscience, An Institute of the KNAW, Amsterdam, The Netherlands; 3 Champalimaud Neuroscience Programme at Instituto Gulbenkian de Ciência, Oeiras, Portugal; Università di Parma, Italy

## Abstract

The study of the neural basis of emotional empathy has received a surge of interest in recent years but mostly employing human neuroimaging. A simpler animal model would pave the way for systematic single cell recordings and invasive manipulations of the brain regions implicated in empathy. Recent evidence has been put forward for the existence of empathy in rodents. In this study, we describe a potential model of empathy in female rats, in which we studied interactions between two rats: a witness observes a demonstrator experiencing a series of footshocks. By comparing the reaction of witnesses with or without previous footshock experience, we examine the role of prior experience as a modulator of empathy. We show that witnesses having previously experienced footshocks, but not naïve ones, display vicarious freezing behavior upon witnessing a cage-mate experiencing footshocks. Strikingly, the demonstrator's behavior was in turn modulated by the behavior of the witness: demonstrators froze more following footshocks if their witness froze more. Previous experiments have shown that rats emit ultrasonic vocalizations (USVs) when receiving footshocks. Thus, the role of USV in triggering vicarious freezing in our paradigm is examined. We found that experienced witness-demonstrator pairs emitted more USVs than naïve witness-demonstrator pairs, but the number of USVs was correlated with freezing in demonstrators, not in witnesses. Furthermore, playing back the USVs, recorded from witness-demonstrator pairs during the empathy test, did not induce vicarious freezing behavior in experienced witnesses. Thus, our findings confirm that vicarious freezing can be triggered in rats, and moreover it can be modulated by prior experience. Additionally, our result suggests that vicarious freezing is not triggered by USVs per se and it influences back onto the behavior of the demonstrator that had elicited the vicarious freezing in witnesses, introducing a paradigm to study empathy as a social loop.

## Introduction

The study of the neural basis of empathy has received a surge of interest in the last years following the description of brain activity in humans that suggests that the representations of a subjects' own emotional states and sensations are partially activated when witnessing the disgust, pain or pleasure of others [1], [2], [3], [4], [5], [6], [7], [8], [9], [10], [11], [12]. In particular, this evidence has been taken to suggest that a neural mechanism, similar to the mirror neurons found in the ventral premotor and inferior parietal lobe of the monkey, which respond both during the execution of goal directed actions and the observation of the same actions executed by others [13], [14], [15], [16], [17], [18], [19], [20], could be at work in emotional and somatosensory brain circuits as well [21], [22]. Testing this idea would require single cell recordings and experimental manipulations of the brain regions involved in empathy. Such invasive techniques are not readily applicable in humans (but see [1], [23]); therefore an animal model of emotional empathy would be essential to further our understanding of empathy.

It has been proposed that empathy exists in social animals because the detection of discomfort, distress or fear in conspecifics carries information of high survival value [24]. In the context of developing an animal model of empathy, here we will focus on whether rats and mice, the two most readily available laboratory mammals, show such social transmission of distress cues. Social transmission of information in rats does occur in a wide range of behaviors such as food preference [25], [26], motor [27], [28] and avoidance behaviors [29]. Moreover, rats, can respond with fear and learn from fear reactions of others; for instance, a neutral stimulus can acquire aversive value after an observation of conditioned responses of another rat [30], [31], [32]. Additionally, interactions with a distressed conspecific seem to recruit the amygdala that is also active when experiencing first hand distress [33]. Also mice show evidence of similar social transmission: the observation of a conspecific being shocked has been shown to induce vicarious freezing in mice [34] and to enhance subsequent fear learning in this species [35]. Vicarious behavior in mice seems to be regulated by the degree of relatedness between the interacting individuals [34], [36]. Together, these evidences suggest that rodents are sensitive to what happens to other rodents. Rodents might therefore provide a powerful animal model for studying and manipulating the neural mechanisms of empathy.

In the effort to develop animal models of empathy, it is important to determine what aspect of empathy can actually be modeled. Current conceptualizations of empathy define it as being composed of two components/processes. First, if an individual has an affective reaction that resembles that of another and is triggered by perceiving or imagining the state of that other individual, the individual is said to experience ‘emotional contagion’ [37], [38]. Emotional contagion occurs early in human development: babies are more likely to cry if they hear other babies cry. Second, if that individual is also aware of the fact that its emotional reaction is triggered by that of another, it experiences true empathy. This distinction is important, because empathy proper is more likely to trigger prosocial behavior than emotional contagion. In animals, it is however often impossible to assess whether they are aware of the source of their emotions, and accordingly to disentangle models of emotional contagion from models of empathy.

Since empathy in humans has been shown to be modulated by experience (see Refs. [21], [24], [37], [39] for reviews) in this study we aimed at establishing a paradigm to study both empathy/emotional contagion itself and its modulation by prior experience. A vast number of studies in the literature reported gender differences in empathy and social perception [40], [41], [42], [43], [44], [45], moreover, gender differences in social modulation of behaviors have been reported in rats [46], [47], [48] with stronger effects in females. We therefore use female rats in this study.

In Experiment 1, we examined the behavior of two interacting female rats while one of them, the demonstrator, experiences a series of unconditioned aversive stimulus (5 footshocks) while the other, the witness, can hear, see and smell the reaction of the demonstrator. To investigate whether prior experience with a similar aversive stimulus would modulate the reaction of the witness, we compared the behavior of witnesses that had previously experienced footshocks with that of witnesses that had not. Finally, we also quantified the relationship between the witnesses' behavior and that of the demonstrators to examine if the way that the witness responds to the behavior of the demonstrator might in turn influence the behavior of the demonstrator.

We predicted that witnessing the distress reactions of the demonstrator would alter the behavioral pattern of the witness and make the witness' behavior resemble that of the demonstrator, for instance by showing an increased freezing or by expressing other distress-related behaviors. Moreover, we expected such vicarious fear responses to be more pronounced in witnesses that had previously experienced footshocks. Finally, it is reported that rats, when paired with a conspecific, express less conditioned fear responses, suggesting the existence of social buffering effects [49]. Furthermore, the stress status of the partner plays an important role in social buffering effects, e.g. a non-shocked partner (not pre-exposed to footshocks) is more effective in attenuating fear responses than a shocked partner (pre-exposed to footshocks) [49]. These findings led us to expect that demonstrators paired with naïve witnesses show less distress than those paired with experienced witnesses, because of the differential social buffering by their paired witness group.

Next, we set out to explore the contribution of various components of the auditory channel in triggering the vicarious freezing in the experienced witness rats. It is well documented that rats emit ultrasonic vocalizations (USVs) and that the frequency and temporal pattern of such vocalizations are determined by specific environmental factors [50], [51], [52], [53]. USVs have been thought to play an important role in the communication between conspecifics but their exact function remains unclear. It has been proposed that they can serve to: localize conspecifics, transfer emotionally valenced information across conspecifics and warn other individuals of external dangers to promote escape or dispersion. (see Refs. [51], [52], [53] for review). Furthermore it has been previously shown that rats emit USVs at a certain frequency (∼22 kHz) in aversive conditions (e.g. during fear conditioning) and in the presence of cues that predict danger [54], [55]. Additionally, a recent study showed that USVs can modulate social transmission of fear in rats [32], however not many studies in the literature examined the role of USVs in potential empathy paradigms. Thus, we set out to test the role of USVs in our potential model of empathy. First, we recorded the USVs produced during the social interactions in Experiment 1 in order to establish the degree of communication between witness and demonstrator pairs. Second, in Experiment 2, we used these recorded vocalizations and played them back to separate groups of naïve and experienced animals while monitoring their behavior, freezing in particular. We produced two kinds of auditory stimuli from the recordings of Experiment 1: i) 22 kHz ultrasonic vocalizations (all other recorded sound were filtered out) ii) 2–4 kHZ control sound that share same temporal characteristics with USVs.

## Materials and Methods

### 1. Subjects

Female Long-Evans rats (250–300 g) from Harlan US Davis were kept in a temperature controlled (22 °C) room and maintained on a reversed 12-h light: 12-h dark cycle (07:00 lights off - 19:00 lights on). Rats were socially housed as 2–4 rats per cage and had *ad libitum* access to food and water. All experiments are conducted during the dark cycle between 09:00 and 13:00 h. All experiments were conducted in strict accordance with the European Community's Council Directive (86/609/EEC) and all experimental procedures were approved by The Institutional Animal Care and Use Committee of the University of Groningen (IACUC-RuG, approval number: 4669).

### 2. Experiment 1

#### 2.1. Groups

Adult female rats were randomly assigned to one of the witness or one of the demonstrator groups, each witness and demonstrator pair is composed of cage-mates and therefore housed together from arrival till the end of the experiment. Witnesses and demonstrators were divided into the following subgroups: Witness groups - Naïve Control Witness, Naïve Shock Witness, Experienced Control Witness and Experienced Shock Witness; Demonstrator groups - Demonstrator paired with Naïve Control Witness, Demonstrator Paired with Naive Shock Witness, Demonstrator paired with Experienced Control Witness, Demonstrator paired with Experienced Shock Witness (see table 1 for the explanation and abbreviations of the experimental groups and pairs). Rats were handled and habituated 3 minutes to the experimenter everyday for 10 days preceding the experiment. All rats were habituated to the transportation and experimental room for 20 minutes/day for 3 days prior to the experiment.

**Table 1:** Conditions and Groups in Experiment 1.

Condition	Witness	Demonstrator
**Control**	NcWNaive control Witness	D(NcW)
	EcWExperienced control Witness	D(EcW)
**Shock**	NsWNaive shock Witness	D(NsW)
	EsWExperienced shock Witness	D(EsW)

Each row indicates the Witness group and its paired Demonstrator group.

#### 2.2. Apparatus

To ensure that experienced witnesses could be familiarized with footshocks prior to the Empathy Test without generating conditioned fear for the context of the Empathy Test, two different chambers (context A and B) were used for the Pre-Exposure and Empathy Test in a counterbalanced fashion. Each chamber consisted of two adjacent animal compartments - witness compartment and demonstrator compartment (each D24 cm x W25 cm×H34 cm) divided by a perforated transparent Plexiglas divider. The dimensions of the two chambers were identical but the two contexts (A and B) were modified to maximize their discriminability by the animals. Context A had metal-coated sides, a transparent front door and lid, and was illuminated using a dim red light. Context B had side panels coated with a striped pattern using latex-based colors, a patterned solid front door and lid, and was illuminated using a bright white light. In both contexts (A and B), the demonstrator area had a stainless steel rod floor to deliver shocks while a solid Plexiglas sheet covered the witness area's floor. The demonstrator area of each chamber (context A or B) was used for the Pre-Exposure training of the experienced witnesses. Experienced witnesses that received the footshock in context A, were then tested in context B in the Empathy Test or vice-versa. Between animals, chambers were wiped twice with different substances to ensure the contexts differed in odor: context A- 70% alcohol and then 3% mint soap solution and context B- 3% vinegar and then antibacterial soap solution.

#### 2.3. Pre-Exposure

**Figure 1:**
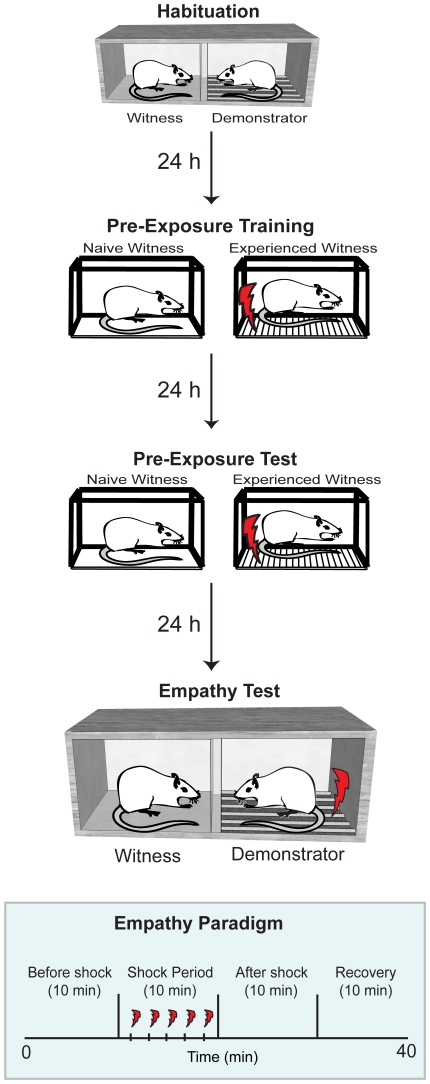
Illustration of experimental design of Experiment 1. Pairs of rats were exposed to the Empathy Test context for 15 minutes (Habituation). Twenty-four hours later, witnesses were placed in the other context, and either received or not a number of footshocks (Pre-Exposure Training). Twenty four hours later, the witnesses were tested for long-term retention of this experience by replacing them in the pre-exposure context and measuring freezing (Pre-Exposure Test). Twenty-four hours later, demonstrator - witness pairs were placed again in the 2 compartments of the Empathy context. This time, the demonstrator (right) receives shocks through the floor grid while the witness (left) can hear, see and smell the demonstrator through a perforated Plexiglas dividing screen. The lowest panel schematizes the time course of the Empathy Test session.

All witnesses were placed in the Pre-Exposure environment individually and after 15 minutes of exploration, only experienced witness groups received 4 footshocks (1 second each, 0.8 mA) separated by random intervals ranging between 240 and 360 seconds (Fig. 1). The pre-exposed rats were housed individually for 1 h after pre-exposure before being returned to their home cages. Twenty-four hours later, both naïve and experienced witness rats were individually tested in the same Pre-Exposure context for 5 minutes (this session will be referred as Pre-Exposure test) and freezing behavior was scored during the last 3 minutes. As this test session could lead to extinction of the acquired fear, at the end of the 5 minutes of Pre-Exposure test, experienced witnesses received one reminder footshock (1 second, 0.8 mA) before they were taken out of the chamber. Again, the rats were then housed individually for 1 h before returning to their home cage.

#### 2.4. Empathy Test

All witness-demonstrator pairs were habituated to the Empathy Test environment a day prior to the Pre-Exposure training of witnesses. In the Empathy Test, the witness and demonstrator constituting a pair were placed in the two adjacent areas of the Empathy Test chamber for a total time of 40 minutes (Fig. 1). After 10 minutes of baseline, in the shock condition, five footshocks (each footshock 5 seconds, 0.8 mA) separated by random intervals of either 2 or 3 m, were delivered to the demonstrator rat only (Fig. 1). In the control condition, the exact same procedure was used, except that a Plexiglas floor separated the demonstrator rat from the metal grid through which the shocks were delivered. This ensured that any sounds or vibrations generated by the shock device would be identical between the shock and control conditions, but the actual footshocks would only reach the experimental but not the control demonstrators. Any differences in freezing rate between the two conditions therefore cannot be due to classical conditioning to the sound of the shock device. After the last shock delivery, rats were left in the box for an additional 20 minutes.

#### 2.5. Behavioral Scoring and Analysis

**Figure 2:**
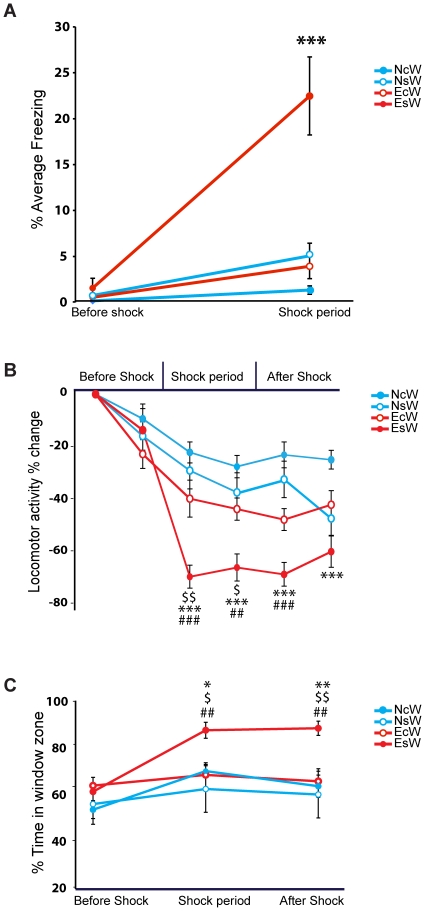
Behavior of 4 witness groups in Empathy Test. Naïve control witness (NcW), experienced control witness (EcW), naïve shock witness (NsW), experienced shock witness (EsW). (A) % Average freezing before shock and during shock period by witnesses. ***p<0.001 EsW compared to all the other witness groups. (B) % Change in locomotor activity before shock, shock and after shock periods. % Change in locomotion is relative to the first time bin that served as baseline and thus has a value of zero by definition. $p<0.05, $$p<0.01 EsW compared to EcW; ##p<0.01, ###p<0.01 EsW compared to NcW; ***p<0.001 EsW compared to NsW. (C) % Time spent in window zone by witnesses. $p<0.05, $$p<0.01 EsW compared to EcW; ##p<0.01 EsW compared to NcW; *p<0.05, **p<0.01 EsW compared to NsW. All data is presented as mean ± S.E.M (n = 11–15 per group).

The entire test sessions were videotaped with a CCD black and white camera (Model SSC-M370 CE, Sony, Japan) mounted on the chamber and connected to an MPEG-encoder PC. Movies were stored in MPEG-2 digital format for later behavioral scoring. Live image from the same camera was transferred to a PC running a video-tracking system (Ethovision 3.1; Noldus information technology, Wageningen, Netherlands) for quantification of general movement and locomotor activity of the witness groups. Locomotor activity of witnesses is sampled as 5 minute time-bins and the percentage change in locomotion was calculated by subtracting the locomotor activity measured in the first 5 minutes (taken as a baseline) from the locomotor activity sampled in the subsequent 5 minute time-bins (in total 8 time-bins were used: 1^st^ and 2^nd^ -before shock, 3^rd^ and 4^th^ -shock period, 5^th^ and 6^th^ -after shock and 7^th^ and 8^ th^ recovery period, Fig. 2b illustrates only the first 6 time-bins). Additional video-tracking analysis was run to quantify the amount of time spent by witnesses in close distance to the demonstrator. For this analysis, the observer's compartment was divided in a far and a close half, relative to the screen dividing the two rats (each zone is 12.5 cm wide) and time spent in the zone close to the demonstrator's compartment (window zone) is calculated in three 10 minute time periods, each corresponding to before shock, shock and after shock periods, respectively.

Freezing behavior was scored in the Pre-Exposure training, Pre-Exposure test and in the Empathy Test sessions. A trained researcher that was blind to the experimental condition, performed the behavioral scoring from the digital movies using Observer XT (Noldus information technology, Wageningen, Netherlands) and Jwatcher (http://www.jwatcher.ucla.edu/). A rat was considered to be freezing if it was (i) in the stereotypical crouching posture and (ii) not moving except for respiration related movements. In the Empathy Test, total time of freezing scoring consisted of 14 minutes divided in 6 time-bins per rat. The first time bin lasted from -2 minute to 0 relative to the onset of the first shock. The other five time-bins corresponded to the time following each of the 5 shock trials (since the inter-shock interval was either 3 or 2 minutes, the time-bins used corresponded to 3 time-bins of 2 minutes and 2 time-bins 3 minutes). For control groups, the same scoring schedule was used. Freezing scores were calculated as the percentage of time during each bin that the rats spent freezing. Average percentage freezing in shock period was calculated by averaging the freezing scores in 5 time bins following the footshock trials.

#### 2.6. Ultrasonic Vocalization Recordings and Analysis

Sounds were recorded with a high-frequency omnidirectional microphone (Earthworks M30, frequency range 5–30 kHz, Earthworks Inc., Milford NH) mounted on the chamber, and amplified (Edirol FA-66, Roland Corporation, Los Angeles, CA). Sounds were digitized at 96 kHz, 16 bits and stored in wav format using Adobe Soundbooth CS3 (Adobe Inc.) on a Macintosh computer. In order to count the number of USVs emitted by witness-demonstrator pairs, wav files were processed in Matlab (Mathworks Inc.) to create sound spectrograms using short-time fast Fourier transform (sFFT) with a window of 256 time points and an overlap of 75%, resulting in a final frequency resolution of 1.5 kHz and time resolution of 0.6 ms. Frequencies outside 15–30 kHz were truncated. Time points containing USVs were separated from those containing only environmental noise by considering the standard deviation of the (filtered) power spectrum of each time point.

Time points containing USVs were clearly identifiable as having a higher standard deviation in the power spectrum with respect to time points containing only environmental noise. We therefore set the time points containing only noise to 0 dB, and summed the power of each time point across frequencies. The resulting vector was smoothed with a moving average of 100 time points (corresponding to approximately 66.67 ms) to increase the signal-to-noise ratio. The nonzero time points of this vector were used to calculate the number of calls, and to compute the distribution of the estimated number of calls for different durations. Only the number of emitted USV in the time frame of freezing scores (6 time bins, see behavioral scoring and analysis for details) was taken into consideration. In order to check the accuracy of the algorithm, the number of USVs in the recording of 5 different demonstrator-witness pairs was quantified both by the algorithm and manually. The number of calls detected by algorithm matched the number of manually counted calls during the same interval.

### 3. Experiment 2

#### 3.1. Groups, Chambers and Experimental Design

Rats were handled and habituated 3 minutes to the experimenter everyday for 10 days preceding the experiment. All rats were accustomed to the transportation and experimental room for 20 minutes/day for 3 days prior to the experiment. On the first day of the experiment, all rats were habituated to the Sound Test chamber (D25×W40×H40) for 15 minutes. Then, rats were divided into two groups: Experienced and Naïve. Experienced animals were trained with footshocks according to the Pre-Exposure training schedule described in Experiment 1, whereas the other animals were kept naïve to footshock. On the following day, animals were placed in the Sound Test chamber and Control sounds or USV sounds were played back from a high frequency loudspeaker (Precision 8D Studio Monitor, Tannoy Ltd., Scotland, UK) through the holes in the Plexiglas divider also used in Experiment 1. In pilot experiments, playback loudness was adjusted to lead to the same sound intensities in the chamber of the witness rats as in Experiment 1. Since the distance between the speaker and the animal depends on the place preference of the animal, we set the distance of the speaker such that the maximal distance (45 cm) or minimal distance (5 cm) between rat and speaker corresponded to the maximum or minimum distance between the witness and demonstrator pairs in the Empathy Test. The total duration of the Sound test was 40 minutes, however only the time window of interest is analyzed (see below for detailed explanation).

#### 3.2. Auditory Stimulus and Playback

In this experiment two different sounds (USV and Control sounds) were played back to naïve and experienced rats. In order to prepare the USV stimuli for playback, the sound tracks recorded from the EsW-D(EsW) pairs during Experiment 1 were band-pass filtered in the range between 17 and 25 kHz in Adobe Soundbooth CS3 (Adobe, San Jose, CA). No USVs outside this frequency range were observed. Control sounds were generated from the same sound track recorded in Experiment 1 by using the SOX software (http://sox.sourceforge.net/). USVs in each recorded file were pitched down 35 semitones to a range of 2.6–4 kHz, while intensity and temporal characteristics were preserved. This range for the control sound was selected on the basis of the previous findings in the literature that rat effectively discriminates 4 kHz sounds from USVs [56]. Sound presentation started after 10 minutes of baseline at the point in which the first electroshock was given in the recording session (Experiment 1), so as to lead to a similar timing as in the Empathy Test in Experiment 1 (footshock exposure of demonstrator started after 10 minutes of baseline). In addition to this main auditory experiment (Experiment 2) we also conducted a pilot experiment to explore the contribution of other auditory signals contained in the sound track recorded in Experiment 1. In this pilot experiment, the same rats that had only been exposed to the control sound in Experiment 2 were place in the test chamber once more and exposed to a playback of the unfiltered recording (USV and audible sounds) of the Empathy Test. In Experiment 2 and in the pilot experiment, freezing behavior was scored and analyzed using the same time window as in Experiment 1 but only in 2 time-bins corresponding to 2 minutes before the onset of the playback and 12 minutes during sound playback, respectively.

#### 3.3. Behavioral Scoring and Analysis

Behavioral scoring was performed live with Ethovision 3.1. (Noldus information technology, Wageningen, Netherlands). 20% of the animals were also scored blindly and the correlation coefficient between blind and live scoring was found to be nearly perfect (pearson correlation, r_p_ = 0.96, p<0.05).

### 4. Statistical Analysis

A separate analysis was performed on witnesses' and demonstrators' freezing levels. In both cases, we analyzed between and within group changes in freezing behavior using a two-way mixed effect analysis of variance (ANOVA) with time (before and after shock) as a within factor and group (either witness or demonstrator groups) as a between factor. In the analysis of the dynamics of interaction between demonstrator and witness, we analyzed the freezing behavior of demonstrators and witnesses separately. In both cases, we analyzed changes in freezing behavior using a two-way ANOVA with time (6 time bins) as within factor and group (2 shock groups) as between factor. Planned comparisons were conducted using unpaired t-tests to compare the differences between groups, while planned comparisons using paired t-tests were performed to compare the differences between time bins. A two-way mixed effect ANOVA model was used, with factors for time bins (within) and group (between) for the analysis of locomotor activity of witnesses. Further *post hoc* tests were performed for more detailed comparisons between witness groups and time bins. Similarly, differences in the time spent in the window zone were tested with a two-way mixed effect ANOVA with time bins as within and group as between factors, followed by *post hoc* tests. The p values resulting from the latter two analyses were corrected for multiple comparisons with the Bonferroni method. Pearson's correlation was used to calculate the relationship between freezing of EsW and freezing of D(EsW), and between USV and average freezing of demonstrators and as well as between USV and average freezing of witnesses. In the analysis of USV, the percentage of pairs that emitted USV was calculated and compared between NsW-D(NsW) and EsW-D(EsW) pairs with t-test.

## Results

As we were interested in the effect of prior experience with footshock on vicarious fear, we first verified whether Pre-Exposure training with footshock led to the formation of a long-term memory for the aversive event in experienced witnesses. To this end, we compared the freezing behavior of experienced and naïve witnesses in the Pre-Exposure test. We found that experienced witness rats, that received footshocks on the Pre-Exposure training, froze significantly more than naïve witnesses (36.6±5.2%, vs. 1.2 ± 0.7 % (mean±SEM)) in the Pre-Exposure test (t (20)  =  −3.276, p<0.001). This finding confirmed that a long-term memory of the Pre-Exposure event was formed in the experienced witnesses.

### Vicarious fear

To investigate whether rats display vicarious fear when observing a conspecific receiving footshocks, freezing behavior was compared across witness groups (Fig. 2a). A 4 Groups (NsW, EsW, NcW, EcW) x 2 time period (before shock vs. shock period) mixed effect ANOVA for freezing levels revealed a significant main effect of group (F_3,96_ = 12.519, p<0.0001), time period (F_1,96_ = 45.201, p<0.0001) and interaction of group by time period (F_3,96_ = 14.939, p<0.0001). Following planned comparisons showed that EsW displayed higher freezing levels in the shock period compared to all other witness groups (p<0.0001 compared to NsW, NcW, EcW). These results indicate that in our experiment, rats express vicarious freezing behavior when observing a conspecific being shocked but only when they have had prior experience with footshock.

We also analyzed the locomotor activity of the four witness groups using video-tracking. This data provides an overall measure of the witnesses' locomotor activity throughout the whole Empathy test period (Fig. 2b). A 4 Groups (NsW, EsW, NcW, EcW) x 7 time bins (each consists of 5 minutes) mixed effect ANOVA for locomotor activity, indicated a significant main effect of group (F_3,48_ = 7.84, p<0.0001) and effect of time bins (F_6,288_ = 19.748, p<0.0001), and a significant effect of interaction between group and time bins (F_18,288_ = 2.983, p<0.0001). Further post-hoc analyses pointed out that EsW exhibited a significantly larger reduction of locomotor activity in time bins corresponding to shock period and to after shock period (see Fig. 2b for the significant differences relative to other groups). This confirmed the results derived from the analysis of freezing behavior. Locomotion of the four witness groups reconverged during the last 10 minutes of the Empathy Test when all groups showed a similar level of activity (Data not shown). Additionally, by using the video-tracking system, we could also assess whether the witness rats preferred to be close to the demonstrator during the Empathy test session (Fig. 2c). To this end, we divided the witnesses' compartment in 2 equal zones: a window zone close to demonstrator and a wall zone far from the demonstrator. A 4 Groups (NsW, EsW, NcW, EcW) x 3 time periods (before shock, shock, after shock) mixed effect ANOVA comparing the proportion of time spent in the window zone revealed a significant effect of group (F_3, 48_ = 3.063, p<0.05) and effect of time period (F_2, 96_ = 26.394, p<0.0001) and as well as significant effect of interaction between group and time period (F_6, 96_ = 5.846, p<0.0001). Following post hoc comparisons showed that the EsW group spent significantly more time in the window zone close to their demonstrator than all the other witness groups during shock period and after shock period (see Fig. 2c for significant differences relative to other groups).

### Effect of social interaction on freezing behavior of demonstrators

**Figure 3:**
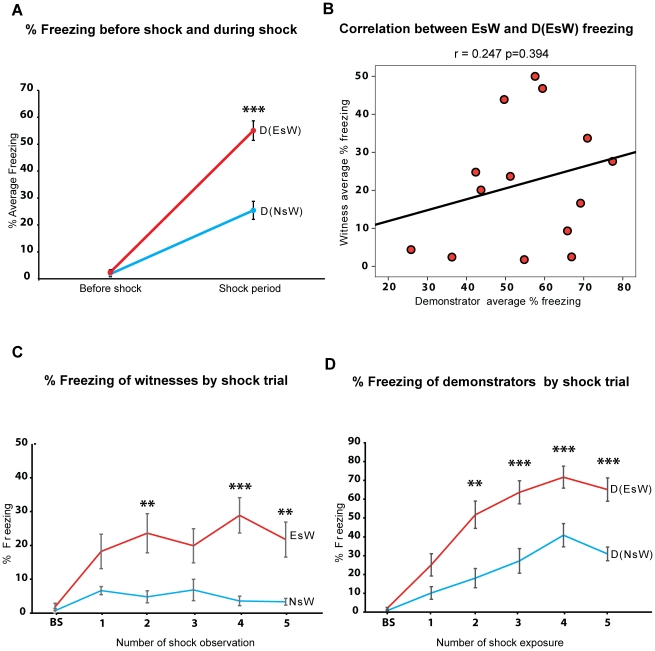
Social modulation of freezing in witnesses and demonstrators. (A) % Average freezing before shock and during shock period by demonstrators paired with naive (D(NsW) and experienced (D(EsW) witnesses. (B) Correlation between freezing levels of experienced shock witness (EsW) and their paired demonstrator (D(EsW)). (C) % Freezing levels of naïve (NsW) and experienced shock witness (EsW) before shock (BS) and during footshock trials (1^st^ to 5^th^). (D) % Freezing of demonstrator group paired with naïve (D(NsW) and experienced (D(EsW)) witnesses before shock (BS) and during footshock trials (1^st^ to 5^th^). **p<0.01, ***p<0.001 compared to respective groups. All data is presented as mean ± S.E.M (n  =  11–15 per group).

A two by two mixed effects ANOVA, demonstrator groups ((D(NsW) vs D(EsW)) and two time periods (before shock and shock period), for freezing levels showed a significant main effect of group (F_1,24_ = 35.619, p<0.0001), of time period (F_1,24_ = 227.615, p<0.0001) and a significant interaction between group and time period (F_1,24_ = 29.890, p<0.0001). Planned comparisons show that before shock trials both groups displayed low levels of freezing that did not significantly differ from each other (p  =  0.658, Fig. 3a), and that footshock delivery led to significantly higher levels of freezing in all demonstrators exposed to footshock (comparison of freezing before shock period vs during shock trials, p<0.0001 for D(NsW), p<0.0001 for D(EsW)). However, D(EsW) expressed significantly more freezing behavior than D(NsW) (p<0.0001, Fig. 3a) during the shock period. To further explore the relationship between freezing displayed by the demonstrator and the witness rats, we examined the correlation between freezing levels displayed by D(EsW) and EsW rats (the group of witness rats which displayed vicarious freezing). We found no significant correlation (Pearson r = 0.247 p = 0.394, Fig. 3b), suggesting that prior experience, rather than differences in freezing displayed by demonstrators (D(EsW) vs. D(NsW)), underlies the differences observed in the behavior of the two shock witness groups.

To further investigate the dynamics of the demonstrator – witness interaction, we conducted analyses to look at the effect of time on the difference of freezing between the demonstrator and witness groups separately (Fig 3c, 3d). A 2 shock witness groups (NsW, EsW) x 6 time bins (before shock, 1^st^ to 5^th^ shock trials) mixed effect ANOVA for freezing behavior indicated a significant effect of group (F_1,24_ = 11.259, p<0.01) and effect of time (F_5,120_ = 3.594, p<0.01). Planned comparisons further unveiled that a significant increase in freezing behavior of EsW relative to the baseline emerged after the 1^st^ footshock trial (p = 0.041 compared to baseline) and that after this initial increase, freezing levels remained stable in the following footshock trials (no difference between 1^st^ shock trial compared to 2^nd^ – 5^th^, P>0.05). Freezing levels of EsW significantly differed from NsW in some of the footshock trials, but the difference was not significant in all cases (Fig. 3c).

A 2 shock demonstrator groups (D(NsW), D(EsW)) x 6 time bins (before shock, 1^st^ to 5^th^ shock trials) mixed effect ANOVA for freezing behavior revealed a significant effect of group (F_1,24_ = 34.585, p<0.00001), effect of time (F_5,120_ = 36.406, p<0.00001) and as well as significant interaction of group and time (F_5,120_ = 4.052, p<0.01). Planned comparisons showed that freezing displayed by both groups of demonstrators (D(NsW) and D(EsW)) increased gradually over footshock trials: Freezing levels of the D(EsW) showed a significant increase on the 1^st^ shock trial relative to baseline (p = 0.004), and increased again after the 2^nd^ shock trial (p = 0.001 relative to the 1^st^). Importantly, the significant difference in freezing levels between D(EsW) and D(NsW) only emerged after the 2^nd^ shock trial and remained significant in the all subsequent shock trials (Fig. 3d). Collectively, these findings show that the differences in freezing between NsW-D(NsW) and EsW-D(EsW) have a different time course for the demonstrators and witnesses. This difference peaked around the 1^st^ shock trial for EsW, but after the 2^nd^ in both demonstrator groups (D(NsW) and D(EsW)).

### Alarm calls during the Empathy Test

**Figure 4:**
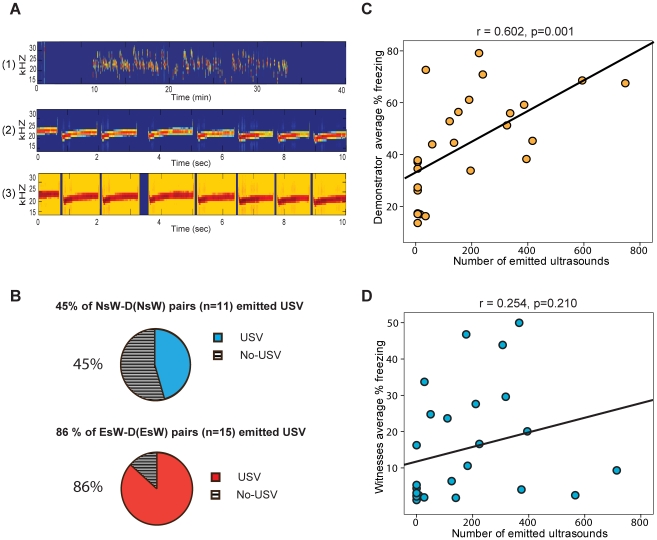
(A) Example sound spectrograms illustrating (1) a 40 min sound track containing USVs recorded in Experiment 1, (2) USVs in a 10 second time window detail, (3) the result of the automated detection of USVs in Matlab, with epochs containing a single 22 kHz-USV shown in yellow. (B) % of naïve shock witness (NsW)-demonstrator (D(NsW) pairs and % experienced shock witness (EsW) and Demonstrator (D(EsW) pairs that emitted USVs. (C) Correlation between the number of emitted USVs and % average freezing response in shock period by both demonstrator groups (paired with naïve shock witness D(NsW) and paired with experienced shock witness D(EsW) together). (D) Correlation between the number of USVs and % average freezing behavior in shock period by naïve shock witness (NsW) and experienced shock witness (EsW) groups.

Analyses of the USVs revealed that not all pairs of rats submitted to shocks emitted USV, and that a larger proportion of EsW-D(EsW) than NsW-D(NsW) pairs emitted USVs (86% versus 45%, p<0.05, Fig. 4b). Separate correlation analysis between the number of USVs emitted and proportion of freezing displayed by witness groups (EsW and NsW) and demonstrator groups (D(EsW) and D(NsW)) show a significant correlation between emitted USVs and mean percentage freezing for the demonstrator groups (r = 0.602, p = 0.001, Fig. 4c), but not for the witness groups (r = 0.254, p = 0.210, Fig. 4d). This shows that differences in the number of USVs emitted by each pair is mainly explained by differences in the freezing behavior of the demonstrators, suggesting that they might be the prime source of USVs.

**Figure 5:**
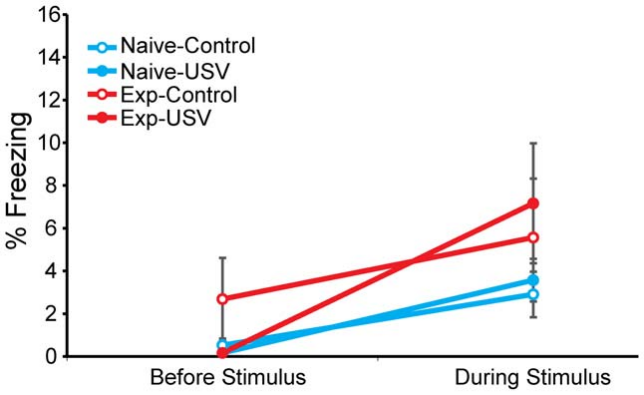
% Freezing behavior of Naïve (Naïve-Control, Naïve-USV) and Experienced groups (Experienced-Control and Experienced-USV) before and during control and USV sound stimulus in Experiment 2. All data is presented as mean ± S.E.M (n  =  10–11 per group).

Next, we examined whether these alarm calls induced freezing in naïve or experienced rats, to which end we performed Experiment 2, a sound playback experiment. Analysing Experiment 2 using A mixed effects ANOVA with freezing as the dependent variable and a 4 groups (Naïve-Control, Naïve-USV, Experienced-Control, Experienced-USV) x 2 time periods (before sound stimulus and during sound stimulus) design revealed a significant effect of time period (F_1,37_ = 18.480, p<0.0001), but no significant effect of group (F_3,37_ = 1.006, p = 0.401) and no significant interaction of group and time period (F_3,37_ = 1.361, p = 0.270). Although there was a significant increase in freezing levels in both experienced and naïve rats during the presentation of any sounds (USV and control sound stimuli), the playback of USVs did not increase the freezing levels above and beyond that of the control sounds in experienced or naïve listeners (Fig. 5).

Finally, to examine if auditory information other than USVs could have triggered freezing in our experiment, we performed a pilot experiment in which we played back the unfiltered recording of Empathy test (USV together with other audible sounds) and we found that the listening rats did displayed freezing behaviour when faced with the combination of USVs and environmental sounds, and that this freezing was stronger in experienced than naïve listeners (t (17)  =  2.177, p<0.05, Fig. S1). Importantly, although these rats were not experimentally naïve, we did not observe any difference in freezing behavior before the onset of the sound stimulus (Fig. S1).

## Discussion

In this study, we describe a paradigm to potentially study empathy in rats and, in particular, the role of prior experience in modulating the empathic response: a demonstrator rat was exposed to footshocks while a cage mate witnesses its distress. We found that demonstrator rats receiving footshocks displayed typical fear responses to this distressing experience, including freezing and emission of USVs and that witness rats that had previously experienced shocks themselves (EsW) displayed similar, albeit less intense, fear responses, including augmented freezing and reduced locomotion. Thus, our experiments confirm that rats can express vicarious fear responses even though not experiencing firsthand pain or distress. This vicarious response was significantly reduced (and no longer significant) in witness rats that had not experienced electroshocks in the past. We further found that the difference in vicarious behavior of the witnesses fed back onto the behavior of the demonstrators that had triggered it in the first place: D(EsW) that were shocked in the company of experienced witnesses progressively froze more than D(NsW) that were exposed to footshocks of the same intensity in the company of naïve witnesses. Finally, the playback of USVs alone did not trigger such vicarious freezing more than control sounds.

### Prior Experience modulates vicarious freezing in rats

Recent studies put forward that mice can display empathic behaviors. In one study, the writhing behavior of a mouse in response to abdominal pain was enhanced if witnessing another mice writhe [36]. The second study showed that mice express freezing when observing a conspecific being shocked [34]. The fact that we found a significant elevation of freezing in EsW rats while observing demonstrator rat receive shocks confirms that a similar form of vicarious distress behavior can be observed in another species of social rodents, the rat. Moreover, that vicarious freezing was lower in NsW compared to EsW adds to our understanding of this phenomenon by showing that having prior experience with footshock can modulate this vicarious reaction. Our findings are in line with the study indicating that conditioned fear responses elicit significant freezing in rats that previously experienced an aversive event but not in naïve rats [32].

The fact that vicarious freezing in NsW was not only lower but also failed to differ significantly from baseline apparently contrasts with the study reporting strong vicarious freezing behavior displayed by naïve mice [34]. Many differences between the two experiments could account for this discrepancy. For example, the intensity and the frequency of the aversive stimulus (footshock) that the witnesses observe seem to play a very important role in modulating empathic responses ([34]supplementary material). Therefore it is reasonable to think that NsW in our experiment might have shown more vicarious freezing if demonstrators had been exposed to more intense or frequent footshocks. Future experiments will be required to determine the adequate intensity and frequency of the footshock to elicit empathic response in naïve witnesses and examine how much prior experience can further augment this response. Moreover, because most other developed empathy models in rodents used male mice [34], [35], [57] whereas in our study we used female rats, it is plausible that there might be species and/or gender differences in vicarious fear behaviors. Species differences have been suggested by studies reported conflicting findings in social modulation of learning between mice and rats. For example, one study indicated that brief social interaction with a recently fear-conditioned conspecific improves the subsequent fear learning in rats [58], whereas similar social interaction impairs fear learning in mice [59]. Gender differences, on the other hand, would dove-tail with gender differences in social support [46] in rats and in social interest in human infant [42] and chimpanzees [43]. Additionally, gender differences in self reported human empathy and in functional activity associated with the human mirror neuron system have been also reported [60], [61]. Nonetheless, it was recently found, with a paradigm somewhat different form the one used in our study, that prior experience plays a crucial role in social transmission of fear between male rats as well [32]. Future experiments testing rats and mice of both sexes in the same paradigms will be necessary to examine the presence of gender and species differences in vicarious freezing.

In our study, we did not examine the effects of the estrous cycle on the vicarious freezing behavior, therefore variance in our data could in part be due to differences in estrous cycle. There is evidence that estrous cycle could affect anxiety and fear responses and therefore affect freezing behavior in female rats [62], [63], [64], however other studies reported no influence of estrous cycle on anxiety levels, fear responses or social interaction in female rats [65], [66].

Other animal studies reported that past experience play a role in reinforcing social transmission of fear and avoidance behavior in rats and empathy in pigeons [29], [30], [32], [67]. Moreover, there is evidence of prior experience dependent modulation of empathic behavior in humans (see Ref. [24] for a review). In particular, functional magnetic resonance imaging studies reported that in humans, hearing piano does not activate the premotor cortex, if one has never played the piano. Five lessons of piano playing, however, are sufficient for the sound of piano to activate areas of the premotor cortex involved in playing the piano [68]. These results have been interpreted as evidence for Hebbian learning: a particular set of sounds (piano notes) becomes associated with a particular inner state (premotor activity required to play the piano) because each time the premotor neurons fire, the participant can hear the consequences of this action, namely the piano notes [69], [70].

Our results are compatible with a Hebbian learning account for the modulation of empathic behaviors by prior experience. When experiencing footshocks, rats will experience their own pain together with the sound and smell of their own reactions (emission of vocalizations, release of pheromones, and sound of running during the shocks alternated with the silence associated with freezing). The sensory consequences of these pain responses could become associated with the experience of pain during footshocks. Once this association is established, perceiving similar sounds and smells while a demonstrator is shocked and reacts accordingly, would trigger, by association, a vicarious form of the first-hand experience of being shocked, including vicarious freezing. Rats that have not experienced this particular type of distress would be expected to have some, albeit weaker associations between the sensory consequences of the demonstrators distress and their own distress. Such weaker associations would originate from the naïve rats experience with other forms of stressors (flying in from the US, grabbing from their home cage, handling by unknown humans etc). These other stressors have probably led to somewhat similar/overlapping behaviors (e.g. squeaking, trying to run away, USVs), that could have been Hebbianly associated with the similar states of distress in these rats. Indeed, in our experiment, there is a trend for NsW to demonstrate more freezing than the NcW.. In addition, because sensing the distress of others is such a valuable source of information about dangers, one might suspect that certain expressions of distress may be inborn triggers of vicarious emotions and behavior, and thereby cause some vicarious freezing without any need for Hebbian learning.

There might however be other, less specific routes for prior experience to influence vicarious freezing. The prior experience of stress in experienced witness groups might have altered their emotional and cognitive state. For instance, the distress during Pre-Exposure could have generated a state of heightened anxiety that would prime these animals to be more sensitive to distress signals in the empathy test or to express their own distress-behavior more readily upon sensing the distress of others [29], [71]. Or, the prior experience might modulate the attentional and motivational states of the witnesses towards the behavior of their conspecifics, including their demonstrators. More attention to the demonstrators would then increase vicarious freezing. In support of that possibility, EsW spent more time close to the demonstrator during and after the shock trials.

One of the core benefits of developing a potential rodent model of experience-dependent empathy is that it will afford us the possibility to disentangle these alternative accounts. For instance, repeating Experiment 1 with the addition of a group that would have experienced a different, but similarly intense, stressor during Pre-Exposure (e.g. immersion in ice water) would be highly instructive: a Hebbian account would predict this new group to freeze less, anxiety or attention accounts, as much, as the electroshock-pre-exposed group.

### Prior experience of witnesses influence the demonstrator's response

We also found that during the shock exposure, D(EsW) expressed more freezing than D(NsW). Given that demonstrator rats were randomly assigned to these two groups and received the exact same treatment throughout the experiment, the only systematic difference between these groups has to originate from systematic differences in the treatment received by their witnesses. The possible explanation for the difference in freezing behaviors of two demonstrator groups might be due to differential social buffering effects by their paired witness groups. Kiyokawa et al showed that the stress status of a partner could influence the social buffering effect in rats. In particular, rats paired with a naïve partner expressed less fear responses in a conditioning context than animals paired with previously shock-exposed partners [49]. Our finding is in line with this observation: demonstrators paired with naïve witnesses showed significantly less freezing responses compared with demonstrators paired with shock pre-exposed witnesses. Issues requiring further study include the channel that is responsible for the influence exerted by the witnesses on the demonstrators and whether the difference in freezing between the demonstrators represents (i) a differences in their distress [53], [55], [69] or (ii) a difference in the propensity to display signs of distress. An analogy to human behavior might clarify these latter alternatives. Would we be genuinely more distressed by a shock if the people around us showed more signs of concern or would we simply be more encouraged to show our distress? Disentangling these possibilities will be an interesting challenge for future research. Importantly, this finding begs us to remember that social interactions are not one-way streets: the demonstrators influenced the witnesses, but the witnesses also influenced the demonstrators. While this conclusion may seem trivial, it actually brakes new grounds in the context of empathy research: most current models of empathy for pain or distress in human neuroscience used prerecorded stimuli [2], [4], [9], [10], [11], [72], [73], [74] or used live interactions but prevented participants from viewing the reactions of their partner [5], [75]. Accordingly, these experiments were unable, by design, to study how the observer's response influences the experience of the demonstrator. Our finding begs us to design experiments in which this feedback-loop and its neural mechanisms can be studied more explicitly in humans as well as in rodents.

The social nature of our experiment is also evidenced by video-tracking data that shows the EsW opted to spend more time in the vicinity of their demontrators than any other witness groups, and by audio recordings that show, the EsW-D(EsW) pairs communicated through more USVs than the NsW-D(NsW) pairs. The fact that the difference in freezing between the demonstrators peaked later than that in the witnesses further suggests that the behaviour of the witnesses could have contributed to that of the demonstrators.

### USV playback alone does not trigger significant vicarious freezing

In the second part of our study (Experiment 2), we examined the contribution of various components of the auditory channel in triggering the vicarious freezing. In both naïve and experienced rats, USVs only produced modest freezing rates (∼5%) that did not exceed the freezing response to control sounds. Therefore, USVs alone cannot account for the bulk of the vicarious stress response in our experiment, where freezing rates reached over 20% in EsW in Experiment 1. Although, the primary function of the rodent USVs remains poorly understood, 22 kHz USV have often been associated with negative and 50 kHz USVs, with positive states [51], [54], [76], [77]. However, it remains unclear whether and when 22 kHz USV can trigger defensive behavior (fleeing or freezing) [54], [76], [78], [79]. At least in our experiment, and with the quality of playback achieved by our equipment, we concluded that USVs playback alone did not produce very robust freezing in naïve or experienced animals. In other situations, USVs might play a more important role [32].

Additionally, in a pilot experiment, by playing back the recorded ultrasounds together with additional audible sounds associated with the behavior of the demonstrator's distress, we observed an experience dependent increase in freezing (Fig. S1). This preliminary finding suggests that audible sounds derived from the fear response of the demonstrator rat might convey distress signals to the witness. In particular the sound of the actions of the demonstrator rat (loud metallic sounds of running intermixed with conspicuous silence) might play an important role in this communication. In monkeys and humans, the sound of the actions of one individual triggers activity in premotor and somatosensory cortices of the listener that mirrors the activity in those of the first individual [15], [16], [73], [80]. Whether similar mirror mechanisms are at work in the rat remains to be explored.

Given that previous studies have shown that visual [28], [36] and olfactory cues [81] can also play a role in social communication in rodents, our pilot data suggests that social modulation and empathy seem to be a multimodal phenomenon, with the dominant modality likely to vary from paradigm to paradigm.

### Conclusions

In conclusion, placing two rats in adjacent compartments and exposing one of the two to footshocks is a simple and viable paradigm to study the way in which distress reactions of a rat influences the behavior of the other rat. Additionally, prior experience of footshocks increases the propensity of a rat to freeze in response to the distress of another. Our paradigm also evidences that the vicarious freezing of the witnessing rat can in turn influence the behavior of the demonstrating rat, closing the social loop.

As mentioned in the introduction, emotional contagion refers to cases in which an emotion in one individual triggers a similar emotion in another, while empathy proper requires that the other is aware of the fact that the triggered emotion is not his/her, but that of another person. Because it is impossible to assess whether rats have any form of awareness of their own emotions (i.e. have feelings), and of the source that triggered the emotion, it is difficult to equate our results with emotional contagion or empathy [75]. Even the degree to which the witnesses in our experiment only showed similar behaviour to that of the demonstrator or felt the same emotion remains veiled. All we can state is that the witnesses reacted with a typical distress behavior to the distress of another rat, and that this represents a potential model for human empathy.

## Supporting Information

Figure S1 % Freezing behavior of Naïve (N) and Experienced (E) groups before and during the playback of the unfiltered recording (22 kHz USVs and the audible sounds <20 kHz) from the EsW-D(EsW) pairs in Empathy Test. All data is presented as mean ± S.E.M (n  =  9–10 per group). *p<0.05, Experienced group compared to Naive.
(TIF)
