# Supplementary figures and images for: Experience Modulates Vicarious Freezing in Rats: A Model for Empathy
Source: PLoS One. 2011 Jul 13;6(7):e21855. doi: 10.1371/journal.pone.0021855 (PMC3135600; doi:10.1371/journal.pone.0021855)

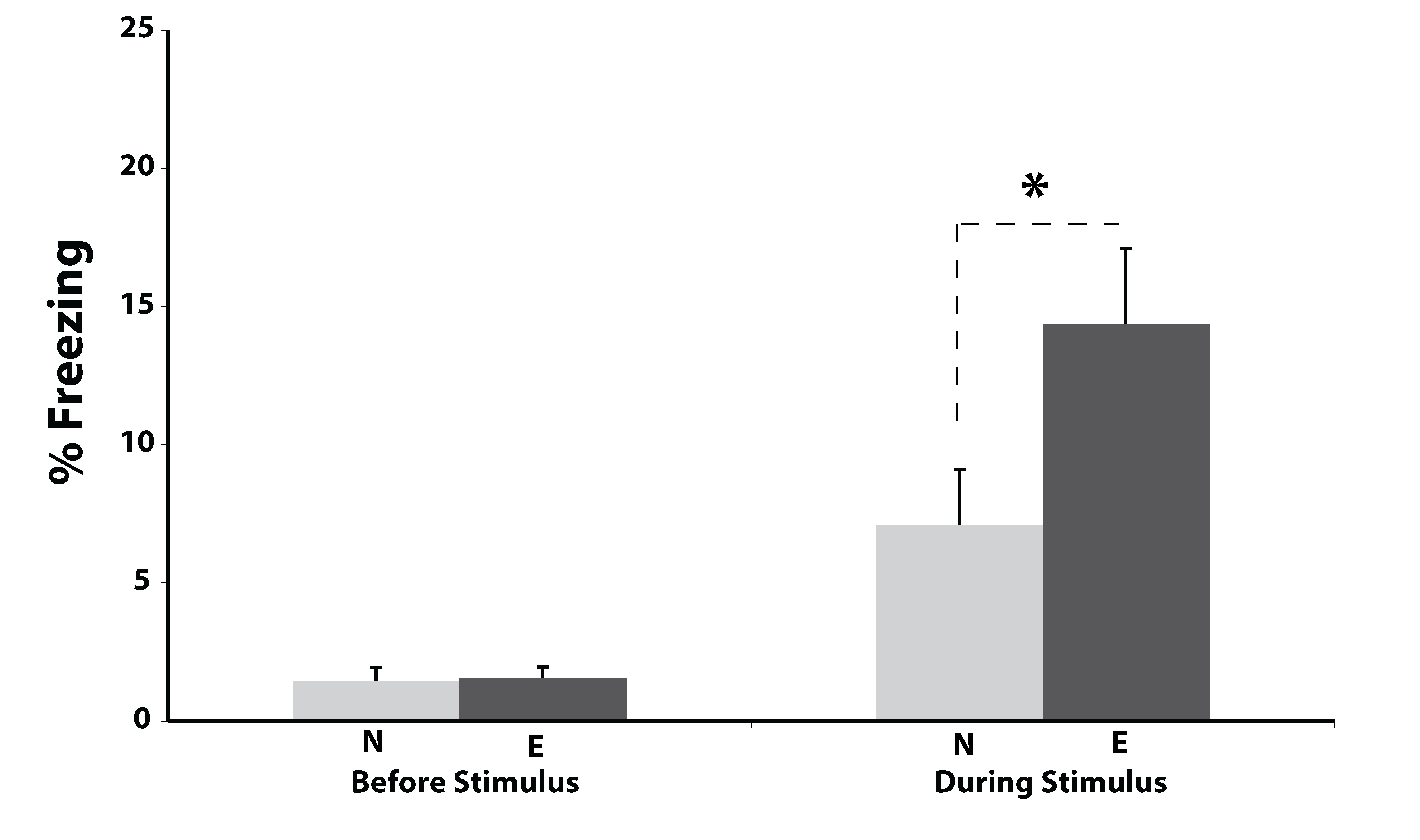

Supplement: Figure S1 — % Freezing behavior of Naïve (N) and Experienced (E) groups before and during the playback of the unfiltered recording (22 kHz USVs and the audible sounds <20 kHz) from the EsW-D(EsW) pairs in Empathy Test. All data is presented as mean ± S.E.M (n = 9–10 per group). *p<0.05, Experienced group compared to Naive. (TIF) [file pone.0021855.s001.tif]
